# Hepatocellular Carcinoma Recurrence as Isolated Adrenal Metastasis

**DOI:** 10.14309/crj.0000000000001065

**Published:** 2023-06-05

**Authors:** Fadl A. Zeineddine, Zunirah Ahmed, David Victor, Andrew Farach, Mukul Divatia, Sudha Kodali

**Affiliations:** 1 Department of Internal Medicine, Houston Methodist Hospital, Houston TX; 2 Division of Gastroenterology and Hepatology, Houston Methodist Hospital, Houston, TX; 3 Sherrie and Alan Conover Center for Liver Disease and Transplantation, Houston Methodist Hospital, Houston, TX; 4 Department of Radiation Oncology, Houston Methodist Hospital, Houston, TX; 5 Department of Pathology and Genomic Medicine, Houston Methodist Hospital, Houston, TX

**Keywords:** hepatocellular carcinoma, adrenal metastasis, stereotactic body radiotherapy

## Abstract

Hepatocellular carcinoma is the sixth most common cancer in the Western world. The most frequent sites of metastasis are lungs, lymph nodes, and bones. Risk factors for extrahepatic metastasis are advanced intrahepatic lesions, vascular invasion, elevated tumor markers, and viral hepatitis. Isolated metachronous adrenal metastasis occurring after liver transplantation is exceedingly rare.

## INTRODUCTION

Hepatocellular carcinoma (HCC) represents the fastest rising cause of cancer-related death in the United States.^1^ HCC accounts for 75%–85% of primary liver cancer, and it is the sixth most common cancer in Western countries.^1,2^ Extrahepatic metastases are found in 10%–15% of patients at time of diagnosis with the most frequent site being the lung (47%) followed by lymph nodes (45%), bones (37%), and adrenal glands (12%).^3^ Local treatment including percutaneous ethanol injection, transarterial chemoembolization (TACE), radiotherapy, and radiofrequency ablation of adrenal metastasis of HCC provides a median survival of 12.8 months.^4,5^ Isolated adrenal metastasis is rare, and literature on managing these is scant. In this report, we describe a case of a patient with primary HCC who was found to have an isolated right adrenal gland metastasis 5 years after orthotopic liver transplantation, who was successfully treated with stereotactic body radiation therapy (SBRT) achieving complete response.

## CASE REPORT

**Figure 1.**
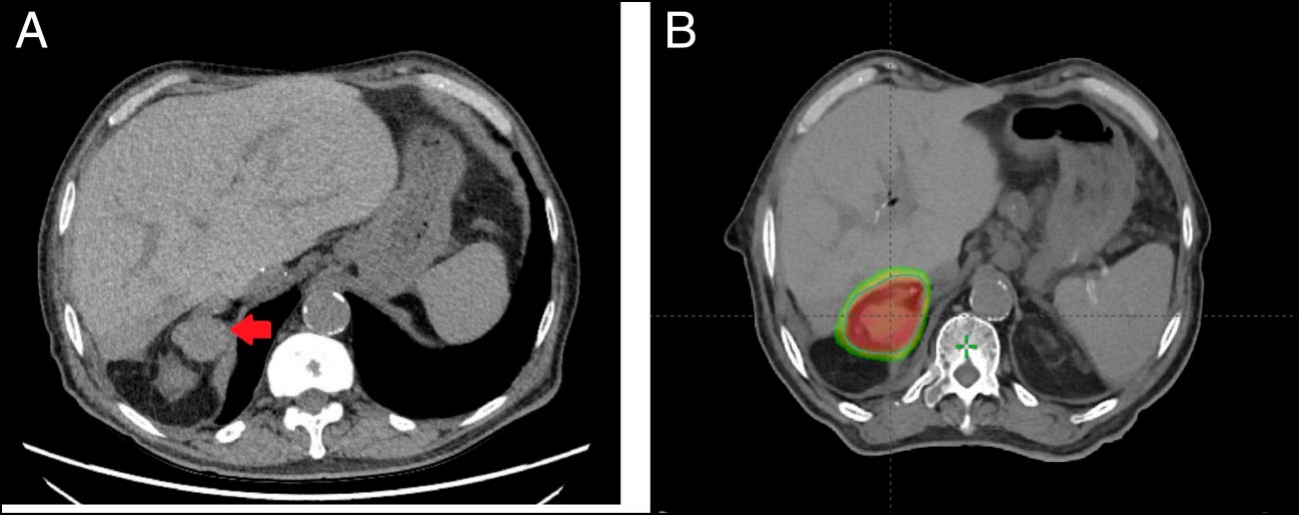
(A and B) Computed tomography of the chest showing the right adrenal mass (arrow).

**Figure 2.**
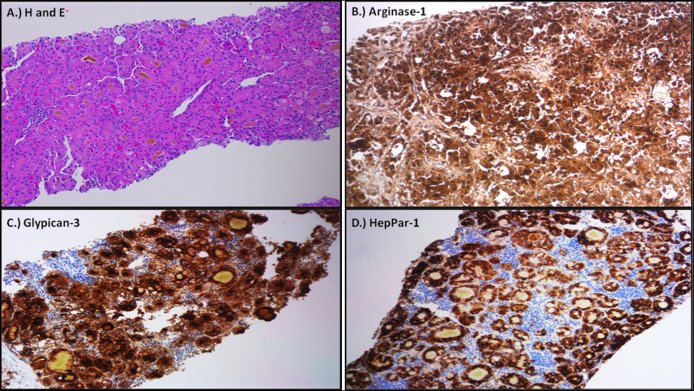
(A) Biopsy of adrenal gland mass demonstrating characteristic features of a moderately differentiated hepatocellular carcinoma exhibiting trabecular and pseudoglandular growth patterns with prominent intratumoral cholestasis (hematoxylin and eosin stain, ×100). (B–D) Immunohistochemical staining for arginase-1, glypican-3, and HepPar-1 corroborates the aforementioned diagnosis with strong expression of these markers in the metastatic tumor cells (immunoperoxidase stain, ×100).

A 72-year-old male patient with medical history significant for cirrhosis secondary to hepatitis C complicated by HCC underwent liver transplantation (LT) in 2016 after TACE. His initial tumor was a 5.8 × 4.3-cm, posterior subcapsular mass in segment 6. There was no evidence of extrahepatic disease. The explant showed cirrhotic liver, with a focally necrotic hepatocellular carcinoma (4.2 × 2.2 × 1.5 cm) demonstrating 80% viable tumor that perforated the visceral peritoneum reaching the Gerota's fascia but not extending to the adrenal gland (ypT4, American Joint Committee on Cancer stage IIIC). Lymphovascular invasion by carcinoma was not identified, and the vascular and bile duct margins were free of tumor. The initial LT was complicated by hepatic artery thrombosis requiring redo liver transplant in 1 week. He completed hepatitis C treatment with ledipasvir-sofosbuvir and achieved sustained virological response. α-fetoprotein measured within 90 days of LT was 136.9 ng/mL. After LT, the patient had good graft function and did well on cyclosporine immunosuppression. The patient's RETREAT score was measured at 3, indicating that he would benefit from imaging surveillance every 6 months for the first 2 years. The patient underwent computed tomography (CT) of the chest, abdomen, and pelvis every 6 months for the first 2 years followed by yearly abdominal/pelvic and chest imaging afterward, which did not show any disease recurrence. In March 2021, 60 months after LT, the patient underwent CT chest for surveillance that showed lobulated solid-enhancing right adrenal mass measuring 4.1 × 3.5 cm, and no abnormality of the left adrenal gland was noted (Figure 1). Previous imaging performed with either magnetic resonance imaging or CT was retrospectively reviewed, and no adrenal admirability was detected. Serum tumor marker levels were as follows: α-fetoprotein level was 13.8, carcinoembryonic antigen 6.6, and carbohydrate antigen 19-9 48. He underwent CT-guided needle biopsy of the adrenal mass. Pathology showed characteristic morphologic features of metastatic moderately differentiated HCC without tumor necrosis in the biopsy specimen. On immunohistochemical staining, the tumor cells strongly expressed arginase-1, glypican-3, and hepPar-1, thus corroborating the diagnosis (Figure 2). Staging scan with CT of the chest and nuclear medicine bone scan was negative, and magnetic resonance imaging of the abdomen showed solitary metastasis to right adrenal gland that was biopsied and no other lesions. The case was discussed in a multidisciplinary tumor board setting, and a decision was made to pursue SBRT with 40 Gy in 5 fractions. Adrenalectomy was deemed high risk because of several comorbidities, including end-stage renal disease on dialysis and cardiac decompensation. Follow-up imaging after completion of SBRT showed response to therapy with complete resolution of the mass. The patient continues to do well and is currently on cyclosporine 75 mg 2 times daily.

## DISCUSSION

Isolated oligometastatic HCC to the adrenal is rare. Our literature review showed that adrenal metastasis occurs significantly later after liver transplantation than after liver resection.^6^ Early recurrence factors include nonanatomical resection, presence of microvascular invasion, and serum α-fetoprotein level ≥32 ng/mL.^7^ In addition, post-transplant immunosuppression can play a contributing role in the acceleration of recurrence.^8^ Teegen et al^6^ identified 6 patients after LT, 3 after liver resection, and 1 after local therapy, who developed adrenal metastasis at a mean of 30.8 ± 19.4 months after diagnosis of primary HCC. Patients with microvascular invasion are significantly more prone to metastatic recurrence.^6,9^ In our case, although the patient did not demonstrate microvascular or macrovascular invasion in the sampled tumor sections, visceral peritoneal perforation of the hepatic capsule was identified on examination of the hepatectomy specimen, and this serves as another factor associated with metastatic spread of HCC to the ipsilateral right adrenal gland. In addition, the lack of vascular invasion on explant pathology may account for the late recurrence period of around 60-month status-post orthotopic liver transplantation. Currently, there are no specific guidelines for treatment of adrenal metastases of HCC. Different therapies including surgical, local ablative methods and systemic therapy have been used. Studies have shown that adrenalectomy provides the most favorable survival outcome with a mean survival time of 112.4 ± 25.2 months and mean time until tumor recurrence was 15.8 ± 3.8 months.^6^ If adrenal metastasis is suspected, tissue diagnosis is required to confirm the metachronous recurrence, and imaging including positron emission tomography scan to rule out other site involvement is recommended before undertaking patient for adrenalectomy.^10^ Owing to our patient's comorbidities, surgery was deemed high risk, and the patient underwent local SBRT. Predictors for unfavorable response to local radiation include multiple intrahepatic tumors, metastasis to additional organ(s), and controlled primary HCC.^11^ Our patient did not have these features. He had a complete response and is still disease-free as of last follow-up 16 months after radiation therapy.

In summary, we present a unique case of delayed recurrence of HCC as an isolated adrenal metastasis that was successfully treated with SBRT. Close surveillance after treatment for HCC for recurrence is imperative for early detection and treatment.

## DISCLOSURES

Author contributions: FA Zeineddine and Z. Ahmed wrote and revised the manuscript. A. Farach revised the manuscript. M. Divatia provided with the pathology images and revised the manuscript. D. Victor and S. Kodali wrote and revised the manuscript. S. Kodali is the article guarantor.

Financial disclosure: None to report.

Informed consent was obtained for this case report.
